# Medical Schools in Scotland

**Published:** 1919-09-06

**Authors:** 


### MEDICAL SCHOOLS IN SCOTLAND.

**Carnegie Trust and the Payment of Class Fees.**—This is a fund to provide under certain regulations eligible students with all or a portion of the class fees of the curriculum. Applicants must be over sixteen years of age, of Scottish birth or extraction, and must have obtained the Leaving Certificate of the Scotch Education Department or recognised equivalent. Forms of application are to be obtained from the Secretary, Carnegie Trust for the Universities of Scotland, 22 Hanover Street, Edinburgh.

### EDINBURGH.

**University of Edinburgh.**—Four degrees in medicine and surgery are conferred by the University, namely, Bachelor of Medicine (M.B.), Bachelor of Surgery (Ch.B.), Doctor of Medicine (M.D.). and Master of Surgery (Ch.M.). The degree of Ch.B. is not conferred on any person who does not at the same time obtain the degree of M.B., and the degree of M.B. is not conferred on any person who does not at the same time obtain the degree of Ch.B. A University diploma is granted in Tropical Medicine and Hygiene (D. T. M. and H.).

The University also grants a Diploma in Psychiatry. The fees for the first and second examinations are £5 5s. each. The first examination comprises anatomy, physiology, pathology, and bacteriology; the second examination, psychology (including experimental psychology), clinical neurology and psychiatry (systematic and clinical). The examinations will be held in March and June.

Courses of instruction for a diploma in public health are being instituted as from October next.

To obtain the Edinburgh medical degree it is necessary to pass the preliminary examination or its recognised equivalent, and to pass four professional examinations. The first is in botany, zoology, physics, and chemistry; the second in anatomy and physiology; the third in pathology, materia medica, and therapeutics; and the final in surgery, clinical surgery, medicine, clinical medicine, midwifery, clinical gynæcology, forensic medicine, and public health. In order to obtain the M.B., Ch.B. degrees, it is necessary to spend at least two of the five years of medical study in the University of Edinburgh. Clinical instruction is given in the Royal Infirmary, which contains nearly 900 beds; Royal Hospital for Sick Children, with 120 beds; Maternity Hospital, with 40 beds available for clinical instruction; City Fever Hospital, with 600 beds, and the Royal Mental Hospital, Morningside, with 500 beds. The total number of beds available for the clinical instruction of students of the University is 2,160. The fees for the four professional examinations amount to £23 2s. Communications should be addressed to the Dean of the Medical Faculty, University New Buildings, Edinburgh, and letters respecting the preliminary examination to Mr. James Dowie, Clerk of Senatus, The University, Edinburgh.

**School of Medicine of the Royal Colleges.** This School of Medicine is constituted by an association of lecturers who lecture in several buildings near the Royal Infirmary. The lecturers are recognised or licensed by the Royal Colleges of Surgeons and Physicians, and also by the University. The teaching is similar to that of the Scottish Universities, and the students receive similar certificates at the close of each session. The lectures qualify for the University of Edinburgh and other Universities; the Royal Colleges of Physicians and Surgeons of Edinburgh, London, and Dublin; the Royal Faculty of Physicians and Surgeons of Glasgow, and other Medical Boards. The whole education required for graduation at the University of London may be taken in this school. The laboratories open and the lectures commence on October 7. The facilities for clinical instruction are similar to those provided for the University. Lectures commence on October 7, 1919. Full particulars and a copy of the calendar of the school may be had gratis from the Dean of the School, 11 Bristo Place, Edinburgh.

### GLASGOW.

**University of Glasgow.**—The whole course of study for the M.B., Ch.B. can be passed in the Medical School of the University. The regulations are similar to those stated above for Edinburgh. Clinical instruction is given at the Western Infirmary, which contains about 600 beds; at the Royal Infirmary, which contains over 600 beds; and also at the Lunatic Asylum, the Eye Infirmary, the Maternity Hospital, the City Fever Hospitals, and various other hospitals. The cost of the course, including matriculation, fees for classes, for hospital attendance, and for professional examinations, amounts to about £150.

Notice is given that, on account of the very large number of first-year students of medicine, including many returned from war service, who were admitted in the summer term 1919, the University is unable to provide accommodation for additional students (other than those matriculated in a previous session) who may desire to begin medical study in October 1919. Accordingly, no further applications for admission to first-year classes can be received until two months before the opening of the summer session on April 21, 1920, when the next five years' course of medical study will normally begin.

**Anderson College of Medicine.**—The college, which provides both Medical and Dental curricula, is situated in Dumbarton Road, adjoining the Western Infirmary and the University. Degrees and diplomas: Certificates of attendance on the lectures are accepted by the Universities of London and Durham, by the Universities of Glasgow, Edinburgh, etc., under conditions stated in the calendars, and by all the Royal Colleges and licensing boards in the United Kingdom. The public-health course qualifies for the Scottish Licensing Board, the Irish Colleges, and the Universities of Oxford, Cambridge, London, etc. The Carnegie Trust extends its benefactions to students of Anderson's College. Particulars may be obtained from Sir W. S. McCormick, LL.D., the Carnegie Trust Offices, Edinburgh. A calendar will be sent on receipt of a postcard by the Secretary to the Medical Faculty, The Anderson College of Medicine, Glasgow, W.

**Queen Margaret College (Women's Department of the University).**—This is an integral part of the University of Glasgow. The courses, regulations, and fees are the same as for men. The instruction is given by University professors and lecturers appointed by the University Court, partly in mixed and partly in separate classes. The College provides a separate building, with class-rooms, laboratories, library, and other teaching appliances. The women have all the rights and privileges of University students. Clinical instruction is provided in the Royal Infirmary and in other hospitals.

Owing to the great number of students seeking admission to the University, it has been found impossible to accept any new first-year medical students in October 1919. Application for admission in April 1920 should be lodged by February 21 1920. The number of women students in the Faculty of Medicine during 1918-19 was 475.

**St. Mungo's College: Medical School of the Glasgow Royal Infirmary.**—The College buildings are situated within the grounds of the infirmary, and are provided with class-room and laboratory accommodation for several hundred students. Students receive their clinical instruction in the wards of the infirmary, which contains 600 beds, and in the Glasgow Royal Maternity and Women's Hospital. The College provides a complete medical curriculum, and the classes qualify for the diplomas of the English, Scottish, and Irish Conjoint Boards, and under certain conditions for the various Universities. Students who have fulfilled the conditions of the Carnegie Trust are eligible for the benefits of this Trust during the whole course of their studies at St. Mungo's College. The classes at St. Mungo's College are open to male and female students equally. Students entering for the Dental diploma can take out all their surgical and medical classes at St. Mungo's College. There is a special department of public health, attendance on which qualifies for the Conjoint Board and the Universities of Oxford and Cambridge. The inclusive fees for the whole Medical curriculum amount to about £100.

**Western Medical School.**—This school, situated in University Avenue, opposite to the principal gate of the University, has been closed during the war.

### ABERDEEN.

**University of Aberdeen.**—The winter session commences on Thursday, October 9, when it is expected that classes will meet in all the ordinary subjects qualifying for graduation in Medicine and Surgery. The regulations are practically the same as those of the other Scottish Universities. Clinical instruction is obtained at the Royal Infirmary, which contains 270 beds, in the Sick Children's Hospital, and several other institutions, including the Royal Lunatic Asylum and the City Fever Hospital. At least two of the five years of study and at least six of twelve specified subjects must be spent or taken in the University. The remaining three years may be spent and the remaining subjects taken in any University of the United Kingdom, or in any Indian, Colonial, or foreign University, recognised for the purpose by the University Court, or in recognised Medical Schools or under recognised teachers. There are four professional examinations, and the cost of Matriculation, class and degree fees for the whole curriculum is about £160. Address, Mr. D. R. Thom, M.A., Secretary, The University, Aberdeen.

### ST. ANDREWS.

**University of St. Andrews.**—Medical students may attend for the first two years of study in St. Andrews (or in Dundee). The remainder of the course must be taken in Dundee, and full particulars respecting the curriculum and examinations may be obtained from Professor Kynoch, Dean of the Faculty of Medicine, the Medical School, Dundee. Clinical instruction is given at the Dundee Royal Infirmary, which has 400 beds (including Maternity Hospital), at the Dundee Eye Institution, the King's Cross Fever Hospital, and the Dundee District Asylum.

